# Evaluation of polygenic risk scores for ovarian cancer risk prediction in a prospective cohort study

**DOI:** 10.1136/jmedgenet-2018-105313

**Published:** 2018-05-05

**Authors:** Xin Yang, Goska Leslie, Aleksandra Gentry-Maharaj, Andy Ryan, Maria Intermaggio, Andrew Lee, Jatinderpal K Kalsi, Jonathan Tyrer, Faiza Gaba, Ranjit Manchanda, Paul D P Pharoah, Simon A Gayther, Susan J Ramus, Ian Jacobs, Usha Menon, Antonis C Antoniou

**Affiliations:** 1 Centre for Cancer Genetic Epidemiology, Department of Public Health and Primary Care, University of Cambridge, Cambridge, UK; 2 Department of Women’s Cancer, Institute for Women’s Health, University College London, London, UK; 3 School of Women’s and Children’s Health, University of New South Wales, Sydney, New South Wales, Australia; 4 Department of Oncology, Centre for Cancer Genetic Epidemiology, University of Cambridge, Cambridge, UK; 5 Centre for Experimental Cancer Medicine, Barts Cancer Institute, Queen Mary University of London, London, UK; 6 Department of Gynaecological Oncology, Barts Health NHS Trust, Royal London Hospital, London, UK; 7 Samuel Oschin Comprehensive Cancer Institute, Cedars-Sinai Medical Center, Los Angeles, California, USA; 8 Department of Biomedical Sciences, Cedars-Sinai Medical Center, Los Angeles, California, USA; 9 The Kinghorn Cancer Centre, Garvan Institute of Medical Research, Sydney, New South Wales, Australia; 10 University of New South Wales, Sydney, New South Wales, Australia; 11 University of Manchester, Manchester, UK

**Keywords:** polygenic risk scores, ovarian cancer, prospective cohort study, risk prediction, evaluation

## Abstract

**Background:**

Genome-wide association studies have identified >30 common SNPs associated with epithelial ovarian cancer (EOC). We evaluated the combined effects of EOC susceptibility SNPs on predicting EOC risk in an independent prospective cohort study.

**Methods:**

We genotyped ovarian cancer susceptibility single nucleotide polymorphisms (SNPs) in a nested case–control study (750 cases and 1428 controls) from the UK Collaborative Trial of Ovarian Cancer Screening trial. Polygenic risk scores (PRSs) were constructed and their associations with EOC risk were evaluated using logistic regression. The absolute risk of developing ovarian cancer by PRS percentiles was calculated.

**Results:**

The association between serous PRS and serous EOC (OR 1.43, 95% CI 1.29 to 1.58, p=1.3×10^–11^) was stronger than the association between overall PRS and overall EOC risk (OR 1.32, 95% CI 1.21 to 1.45, p=5.4×10^–10^). Women in the top fifth percentile of the PRS had a 3.4-fold increased EOC risk compared with women in the bottom 5% of the PRS, with the absolute EOC risk by age 80 being 2.9% and 0.9%, respectively, for the two groups of women in the population.

**Conclusion:**

PRSs can be used to predict future risk of developing ovarian cancer for women in the general population. Incorporation of PRSs into risk prediction models for EOC could inform clinical decision-making and health management.

## Introduction

Ovarian cancer (OC) is the sixth most common cancer in women with 7378 women diagnosed with the cancer in the UK in 2014. Epithelial ovarian cancer (EOC) is the most common type, accounting for 90% of OC, of which two-thirds are serous EOC.^1^ The overall 10-year survival rate for OC is around 36% and is poorer when diagnosed at an advance stage.^2^ Therefore, early diagnosis has the potential to improve survival rates; however, most women with symptoms present with advanced stage disease. Epidemiological studies have estimated the risk of OC in first-degree relatives of patients with OC to be threefold greater than the risk in the general population, indicating the importance of genetic factors in disease susceptibility.^3^ High penetrance mutations in *BRCA1* and *BRCA2* explain around 25% of the observed familial relative risk (FRR)^3^ and a further 10% is explained by moderate-risk mutations in *MLH1*, *MSH2*, *MSH6*, *RAD51C*, *RAD51D* and *BRIP1*.^4^ Genome-wide association studies (GWAS) have identified ~30 common low-risk SNPs that are associated with EOC, accounting for approximately 6.4% of the FRR.^5 6^ Additional potential susceptibility loci were identified by pleiotropy cancer GWAS analysis.^7^ Although individually each SNP is associated with a low risk of EOC, in combination their effects on risk may be greater. Their inclusion in EOC risk prediction models may improve risk precision.^3^ Providing refined personalised cancer risks can result in better risk stratification and hence help in improving early cancer detection and prevention.

Previous published studies investigating the combined effects of EOC SNPs in terms of polygenic risk scores (PRSs) have either been based on retrospective studies or overlapped with the association studies that led to the identification of the SNPs. Here, we use data from an independent prospective population-based cohort study, the UK Collaborative Trial of Ovarian Cancer Screening (UKCTOCS), to evaluate the EOC PRSs in predicting EOC risk prospectively.

## Methods

### Study subjects

UKCTOCS is a randomised controlled trial for OC screening initiated in 2001. Postmenopausal women between age 50 and 74 years were recruited from 13 regional centres in National Health Service (NHS) Trusts in England, Wales and Northern Ireland.^8 9^ Exclusion criteria included self-reported previous bilateral oophorectomy or ovarian malignancy, increased risk of OC due to history of OC or breast cancer in the family or known OC predisposing mutations, or had an active non-ovarian malignancy.^8–10^ All participants provided written informed consent. All women completed a two-page 18-item baseline questionnaire at recruitment which captured data on the known OC risk factors (eg, personal/family cancer history, height/weight, reproductive history, oral contraceptive pill (OCP) and hormone replacement therapy use).^9^ Two postal follow-up questionnaires were sent to the women, with the first 3–5 years post-randomisation and the second in 2014.^8^ A blood sample was donated by each woman at recruitment and serum was extracted as previously described.^9^ Further details on sample processing and DNA extraction are provided in the online supplementary material. Notification of cancer diagnosis and deaths were through NHS Digital for the women residing in England and the Northern Ireland Cancer Registry and Central Services Agency for those residing in Northern Ireland. For women who developed OC, medical notes were retrieved and independently reviewed by an Outcomes Review Committee who assigned histological subtype, stage and grade. For the present study, we used a nested case–control design in women of self-reported white European ancestry. Cases were defined as women diagnosed with incident invasive epithelial ovarian or fallopian tube cancers or primary peritoneal cancer. Two random controls were selected per case, matched on regional centre, age at randomisation and year at recruitment. Following an outcomes review on 31 December 2014,^8^ a total of 750 EOC cases and 1428 controls were included in the present analysis.

10.1136/jmedgenet-2018-105313.supp1 Supplementary data

### SNP selection and genotyping

A panel of 96 SNPs were designed on the basis of their association with EOC risk from the meta-analysis of Kuchenbaecker *et al* 5 (online supplementary figure 1). These included SNPs from 50 regions that demonstrated associations at genome-wide significance level but also regions with associations at p<10^−5^. This was done in view of the ongoing OncoArray experiment^6^ that was being performed on a larger sample size compared with the study of Kuchenbaecker *et al.* For each region, multiple correlated SNPs were selected for inclusion in the panel to ensure data availability in case of SNP genotyping failures.

Genotyping was performed on 96.96 dynamic arrays using the Fluidigm EP1 system (Fluidigm, San Francisco, California, USA) from 10 ng of DNA following the manufacturer’s conditions using the pre-amplification protocol. The 96 SNPs included inventoried and Custom Assay-by-Design TaqMan probes (Applied Biosystems). Analysis was performed using Genotyping SNP Analysis software (Fluidigm). In total, 52 SNPs failed quality control (QC) due to poor clustering on the serum DNA samples, leaving 44 SNPs for analysis. To ensure consistency with the most recent GWAS results, SNPs were selected for inclusion in the PRS if they were in regions that showed genome-wide significance in the OncoArray experiment^6^ (online supplementary figure 1). Of the 44 SNPs, 19 SNPs were from 15 genome-wide significant regions reported in the OncoArray experiment (online supplementary figure 1). In total, 191 samples with call rates <80% were excluded (8%); therefore, 2178 samples passed QC (750 cases and 1428 controls). Any 96-well plates with pass rates <80% were excluded for a particular SNP. Also, 131 duplicate samples were included, and the concordance for duplicate samples was 97.6%. SNPs with significant deviations from Hardy-Weinberg equilibrium were assessed for quality of genotype clustering. All demonstrated clear clusters of genotyping calls and were therefore included in the analysis.

### SNP selection for inclusion in the PRS

The selection of genotyped SNPs for inclusion in the PRS was based on the latest results from the meta-analysis of GWAS for EOC reported by Phelan *et al*.^6^

Two separate PRS were constructed: one for overall EOC and one for serous EOC. The overall PRS was constructed using the set of SNPs that showed associations with overall or any type of EOC at GWAS level. The serous PRS was constructed using the set of SNPs that showed associations with overall or any type of EOC at GWAS level, but also showed associations in the same direction for serous EOC. Only one SNP from each region was used. For each region, if the most significant SNP in the GWAS was among the SNPs genotyped, then it was selected for inclusion in the PRS. If the top SNP was not available, then we used a genotyped SNP (among the 44) from the region which had the highest correlation with the top published SNP. In total, 15 SNPs from 15 regions were included in the overall PRS construction and the same set of SNPs was selected for the serous PRS construction.

### Statistical analysis

To construct the PRS, we first evaluated all pairwise SNP interactions among the SNPs included in the PRS for their associations with EOC risk using logistic regression. In each model, the effects of both SNPs were included (as continuous variables taking values 0, 1 and 2) together with an interaction term between the SNPs. The quantile–quantile plot (qqplot) for all pairwise combinations were examined for the null hypothesis of no interaction effect. A Bonferroni correction was applied to adjust for multiple testing. The adjusted p value threshold was set at 8.3×10^−5^.

The PRS for individual i was defined as

(1) $$PRS_{i}=\beta_{1}g_{1i}+\beta_{2}g_{2i}+...\beta_{k}g_{ki}...+\beta_{n}g_{ni}$$

where g_ki_ is the number of effect alleles for SNP k in individual i (taking values 0, 1 and 2) and β_k_ is the per-allele log odds ratio (OR) for developing EOC associated with each copy of the effect allele of SNP k and n is the total number of SNPs used (n=15, online supplementary table 1). The log OR estimates for each SNP were obtained from the combined COGS and OncoArray association analyses of the Ovarian Cancer Association Consortium^6^ (online supplementary table 1). For the overall PRS, we used the log OR estimates for developing overall EOC; for the serous PRS, we used the log OR estimates for serous EOC. For women with missing SNP genotypes (due to genotyping failures), we used the mean genotypes in controls and cases for each SNP separately.

Logistic regression was used to examine the association between the PRS and outcome. When investigating the overall PRS, the outcome (cases) was all invasive EOC (any histotype). When investigating the PRS for serous EOC, the outcome was serous EOC only. In each case, the PRS was treated as either a continuous or a categorical variable. The PRS was standardised by subtracting the mean in controls and dividing by the standard deviation (SD) in controls. When used as a categorical predictor, the PRS was grouped into the percentiles: [0,5%), [5%,10%), [10%,20%), [20%,40%), [40%,60%), [60%,80%), [80%,90%), [90%,95%) and [95%,100%] on the basis of the PRS distribution in controls with [0,5%) as the lowest 5% PRS group and [95%,100%] as the highest. The middle [40%,60%) group was used as the reference category. The observed ORs by PRS percentiles were compared with the theoretical OR predictions under a multiplicative polygenic model of inheritance.^11^

Additional analyses were performed by adjusting for age and family history of EOC. Age was considered to be the age at EOC diagnosis for cases and at the age at last follow-up or age at the first non-EOC cancer (whichever occurred first) for controls. Two separate family history variables were constructed indicating (1) the number of relatives diagnosed with EOC in first-degree relatives and (2) indicating the number of affected relatives in both first-degree and second-degree relatives. We performed separate analyses adjusting for each of the two family history variables. Family history information was available at the baseline questionnaire. The discriminatory power of the PRS was assessed by the C-statistic using R package ‘pROC’. All statistical tests were two-tailed, and the significance threshold was set at 0.05.

The age-specific absolute risks of developing EOC by PRS percentiles were calculated by considering the OC incidence by PRS percentile and competing causes of mortality (other than EOC). We used data on EOC incidences and mortality rates were from the UK during 2012–2014.^1 12^ The OC risks by PRS percentiles were calculated as

(2) $$Risk_{PRS}\left(t\right)=\sum_{u=0}^{t}\lambda_{PRS}\;\left(u\right)\cdot S_{PRS}\;\left(u\right)\cdot S_{m}\left(u\right)$$

where $\lambda_{PRS}\;\left(t\right)$ is the OC incidence associated with PRS at age t, $S_{PRS}\;\left(t\right)$ is the PRS-specific survival function of being OC free at age t and $S_{m}\left(t\right)$ is the survival function at age t calculated on the basis of incidences of death from causes other than OC. To calculate the OC incidence for each PRS percentile, we assumed that the average, age-specific OC incidences, over all PRS percentiles, agreed with the population OC incidences and calculated the PRS-specific incidence recursively. Details of these methods have been described elsewhere.^11 13 14^

## Results

Data on 1428 controls and 750 EOC cases were included in the overall PRS analysis. Data on the same 1428 controls and 489 serous EOC cases (including 417 high-grade serous) were included in the serous PRS association analysis. Table 1 summarises the study characteristics and provides a breakdown by histology subtype. A summary of the SNPs included the PRS is shown in online supplementary table 1.

**Table 1:** A summary of epidemiological characteristics of the subjects included in the nested case–control study

	Controls	Cases	P_difference*
***Women (n)***	1428	750	
***Age at baseline (%)***			0.70
<60	496 (34.7%)	256 (34.1%)	
60–69	701 (49.1%)	381 (50.8%)	
≥70	231 (16.2%)	113 (15.1%)	
***Age at censoring (%)***			<0.0001
<60	50 (3.5%)	107 (14.3%)	
60–69	388 (27.2%)	354 (47.2%)	
70–79	681 (47.7%)	275 (36.7%)	
≥80	309 (21.6%)	14 (1.9%)	
***Birth cohort (%)***			0.76
<1930	28 (2.0%)	10 (1.3%)	
1930–1939	639 (44.7%)	339 (45.2%)	
1940–1949	669 (46.8%)	354 (47.2%)	
≥1950	92 (6.4%)	47 (6.3%)	
***Mean age at baseline (SD)***	63 (6.2)	63 (6.2)	
***Mean censored age (SD)***	74 (7.1)	68 (6.6)	
***Mean PRS (SD)***			
Overall	−0.47 (0.27)	−0.39 (0.27)	
Serous	−0.55 (0.35)	−0.43 (0.36)	
***Family history of ovarian cancer (%)***			
Considering only first-degree relatives			0.088
Zero affected relatives	1387 (97.1%)	718 (95.7%)	
One affected relative	41 (2.9%)	32 (4.3%)	
Considering both first-degree and second- degree relatives			0.15
Zero affected relatives	1356 (95.0%)	701 (93.5%)	
One or more affected relatives	72 (5.0%)	49 (6.5%)	
***Morphology/histotype (%)***			
Serous		489 (65.2%)	
High grade		417 (55.6%)	
Low grade		23 (3.1%)	
Missing		49 (6.5%)	
Clear cell		29 (3.9%)	
Endometrioid		56 (7.5%)	
Mucinous		24 (3.2%)	
Others		152 (20.3%)	

*χ^2^ tests for differences in the distributions between cases and controls.

### Pairwise SNP*SNP interaction analysis

**Figure 1:**
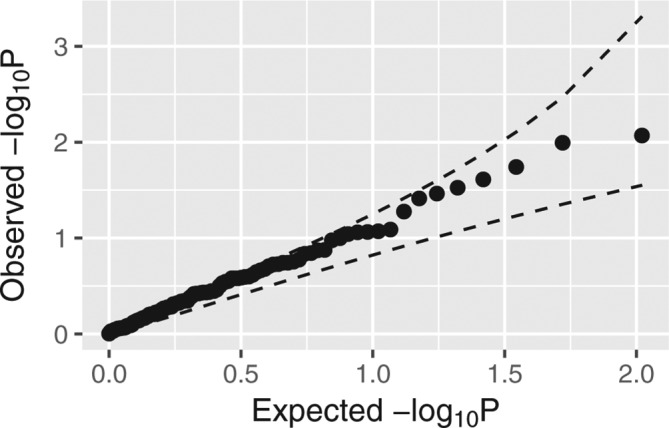
Quantile–quantile plot shows the observed against expected −log_10_p values of pairwise SNP*SNP interaction tests under the null hypothesis of multiplicative model. The dashed line shows the 95% concentration band.

A total of 105 pairwise SNP*SNP interaction tests were performed but there was no significant evidence of interaction between any SNP pairs after a Bonferroni adjustment. The plot of observed against expected −log_10_p values did not show a significant departure from the expected values under the null hypothesis of no interaction (figure 1).

### Association between PRS and ovarian cancer

**Figure 2:**
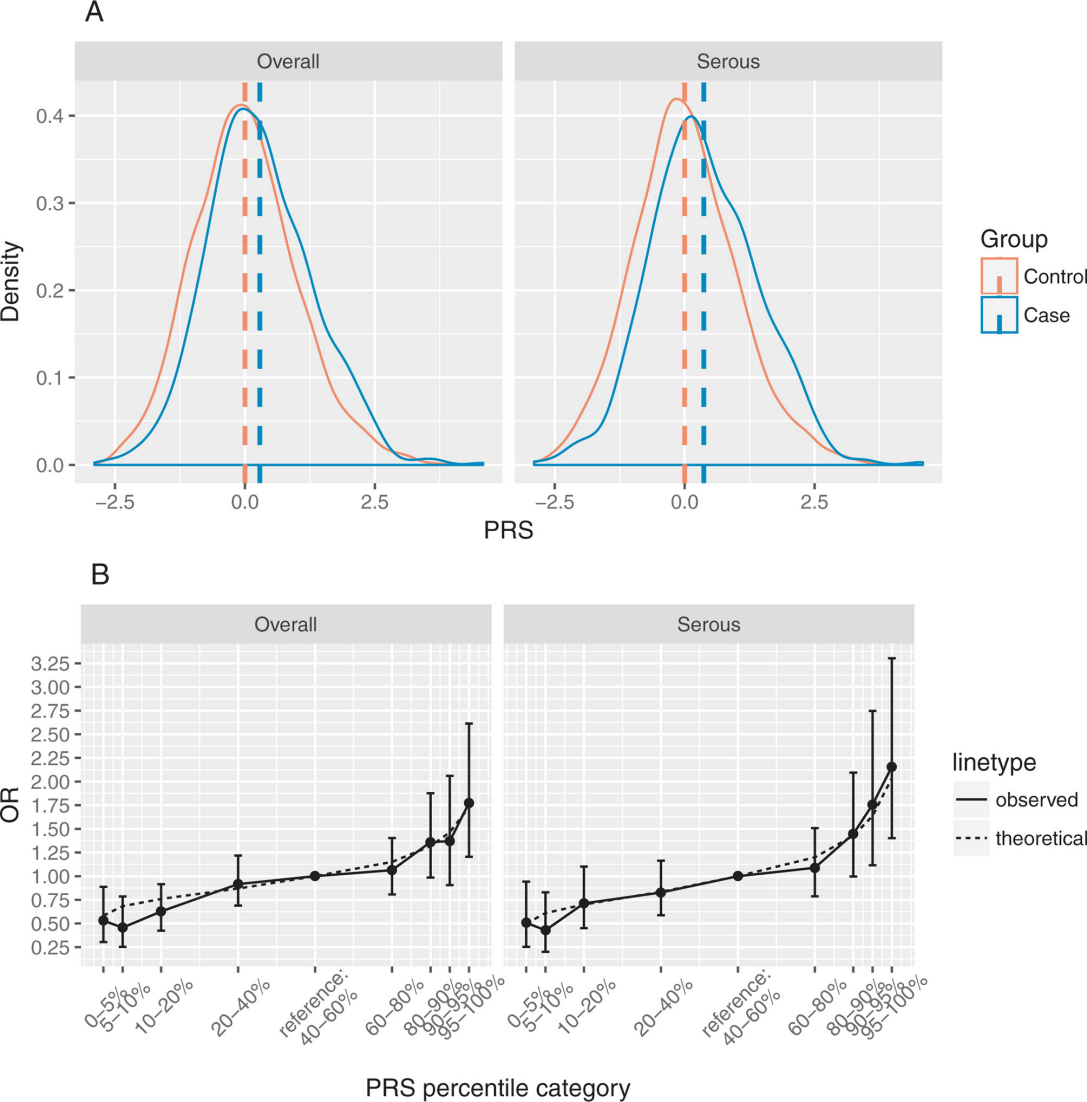
(A) Distribution of the standardised overall and serous polygenic risk scores (PRSs) in overall and serous ovarian cancer cases and controls. The dashed vertical lines show the PRS means. (B) OR estimates between overall/serous PRS percentiles and overall/serous ovarian cancer risk relative to the middle PRS quintile (40%–60%). The solid line shows the estimated ORs with 95% CI, and the dashed line represents the theoretical OR values assuming multiplicative model.

Figure 2A shows that the PRS follows a nearly normal distribution in both controls and cases where the mean of cases was right shifted by 0.28 for the overall standardised PRS and 0.37 for the serous standardised PRS. There was a significant association between the overall PRS and overall EOC in the nested case–control study and the association was stronger between the serous PRS and serous EOC. The OR per unit SD was estimated to be 1.32 (95% CI 1.21 to 1.45, p=5.38 × 10^–10^) for the overall PRS and 1.43 (95% CI 1.29 to 1.58, p=1.28 × 10^–11^) for the serous PRS (table 2).

**Table 2:** Association between polygenic risk scores (PRS) and ovarian cancer in different age groups

Age group	Overall		Serous	
	OR (95% CI)	P values	OR (95% CI)	P values
All ages	1.32 (1.21 to 1.45)	5.38×10^–10^	1.43 (1.29 to 1.58)	1.28×10^–11^
<60	1.29 (0.91 to 1.86)	0.16	1.46 (1.01 to 2.17)	0.05
60–69	1.28 (1.11 to 1.49)	8.99×10^–4^	1.34 (1.14 to 1.59)	4.51×10^–4^
≥70	1.36 (1.20 to 1.55)	3.02×10^–6^	1.47 (1.26 to 1.72)	7.10×10^–7^
Interaction	1.00 (0.99 to 1.02)	0.88	1.00 (0.98 to 1.02)	0.95

In the overall PRS analysis, we used cases of any type of ovarian cancers and in the serous PRS analysis we used cases of serous ovarian cancer.

The associations between the overall PRS and overall EOC, and between the serous PRS and serous EOC, stratified by age are shown in table 2. No significant interaction between age and EOC was observed in either the overall or the serous group (table 2). Discrimination as measured by the C-statistic was equal to 0.58 (95% CI 0.55 to 0.60) for the overall PRS and 0.60 (95% CI 0.57 to 0.63) for the serous PRS.

The estimated ORs by percentiles of PRS compared with the middle quintile ([40%,60%)) are shown in figure 2B. The ORs increased with increasing PRS percentiles for both the overall and the serous PRS. In the overall group, the OR for developing EOC for women in the lowest overall PRS percentile ([0,5%)) was estimated to be 0.53 (95% CI 0.30 to 0.89) and the OR for those in the highest overall PRS percentile ([95%,100%]) was 1.77 (95% CI 1.20 to 2.61) compared with the women in the middle overall PRS quintile. In the serous group, the OR for developing serous EOC was estimated to be 0.51 (95% CI 0.25 to 0.94) for women in the lowest serous PRS percentile ([0,5%)) and 2.16 (95% CI 1.40 to 3.30) for women in the highest serous PRS percentile ([95%,100%]) compared with the women in the middle serous PRS quintile (table 3). The family history of OC alone did not show significant association with EOC risk (OR for family history in first-degree relatives=1.51, 95% CI 0.94 to 2.41; OR for family history in first-degree and second-degree relatives=1.32, 95% CI 0.90 to 1.91). After adjusting by family history of OC, the OR estimates of PRS percentiles remained similar (table 3) but there was some attenuation in the effect of family history in first-degree and second-degree relatives of 3% on the log-scale. The observed distribution of the OR estimates was in line with the ORs theoretical-predicted values under the assumption that all SNPs interact multiplicatively (figure 2B) with all 95% CI for the observed OR estimates containing the theoretical estimates.

**Table 3:** Association between polygenic risk scores (PRS) percentiles and ovarian cancer risk: unadjusted and adjusted by family history of ovarian cancer (FH) in first-degree or in first-degree and second-degree relatives

PRS percentile category (%)	Controls (n)	Cases (n)	OR (95% CI)		
			Unadjusted by FH	Adjusted by first-degree FH	Adjusted by first-degree and second-degree FH
(a) Overall					
[0,5)	72	20	0.53 (0.30 to 0.89)	0.53 (0.31 to 0.89)	0.54 (0.31 to 0.90)
[5,10)	71	17	0.46 (0.25 to 0.79)	0.46 (0.25 to 0.79)	0.46 (0.25 to 0.79)
[10,20)	143	47	0.63 (0.42 to 0.92)	0.63 (0.43 to 0.92)	0.63 (0.43 to 0.92)
[20,40)	285	137	0.92 (0.69 to 1.22)	0.92 (0.70 to 1.23)	0.93 (0.70 to 1.23)
[40,60)	286	150	1	1	1
[60,80)	285	159	1.06 (0.81 to 1.40)	1.07 (0.81 to 1.42)	1.07 (0.81 to 1.41)
[80,90)	143	102	1.36 (0.99 to 1.88)	1.37 (0.99 to 1.89)	1.37 (1.00 to 1.90)
[90,95)	71	51	1.37 (0.91 to 2.06)	1.37 (0.91 to 2.06)	1.37 (0.90 to 2.06)
[95,100]	72	67	1.77 (1.20 to 2.61)	1.79 (1.21 to 2.64)	1.78 (1.21 to 2.62)
FH				1.52 (0.94 to 2.44)	1.28 (0.88 to 1.87)
(b) Serous					
[0,5)	72	12	0.51 (0.25 to 0.94)	0.51 (0.25 to 0.94)	0.51 (0.25 to 0.95)
[5,10)	71	10	0.43 (0.20 to 0.83)	0.43 (0.20 to 0.82)	0.43 (0.20 to 0.83)
[10,20)	141	33	0.71 (0.45 to 1.10)	0.72 (0.46 to 1.11)	0.72 (0.45 to 1.11)
[20,40)	287	78	0.83 (0.59 to 1.16)	0.83 (0.59 to 1.17)	0.83 (0.59 to 1.17)
[40,60)	286	94	1	1	1
[60,80)	285	102	1.09 (0.79 to 1.51)	1.09 (0.79 to 1.51)	1.09 (0.79 to 1.51)
[80,90)	143	68	1.45 (1.00 to 2.10)	1.45 (1.00 to 2.10)	1.45 (1.00 to 2.10)
[90,95)	71	41	1.76 (1.12 to 2.75)	1.77 (1.13 to 2.77)	1.77 (1.12 to 2.77)
[95,100]	72	51	2.16 (1.40 to 3.30)	2.15 (1.40 to 3.30)	2.14 (1.39 to 3.28)
FH				1.54 (0.88 to 2.63)	1.36 (0.88 to 2.08)

### Absolute risk of developing OC by PRS percentiles

**Figure 3:**
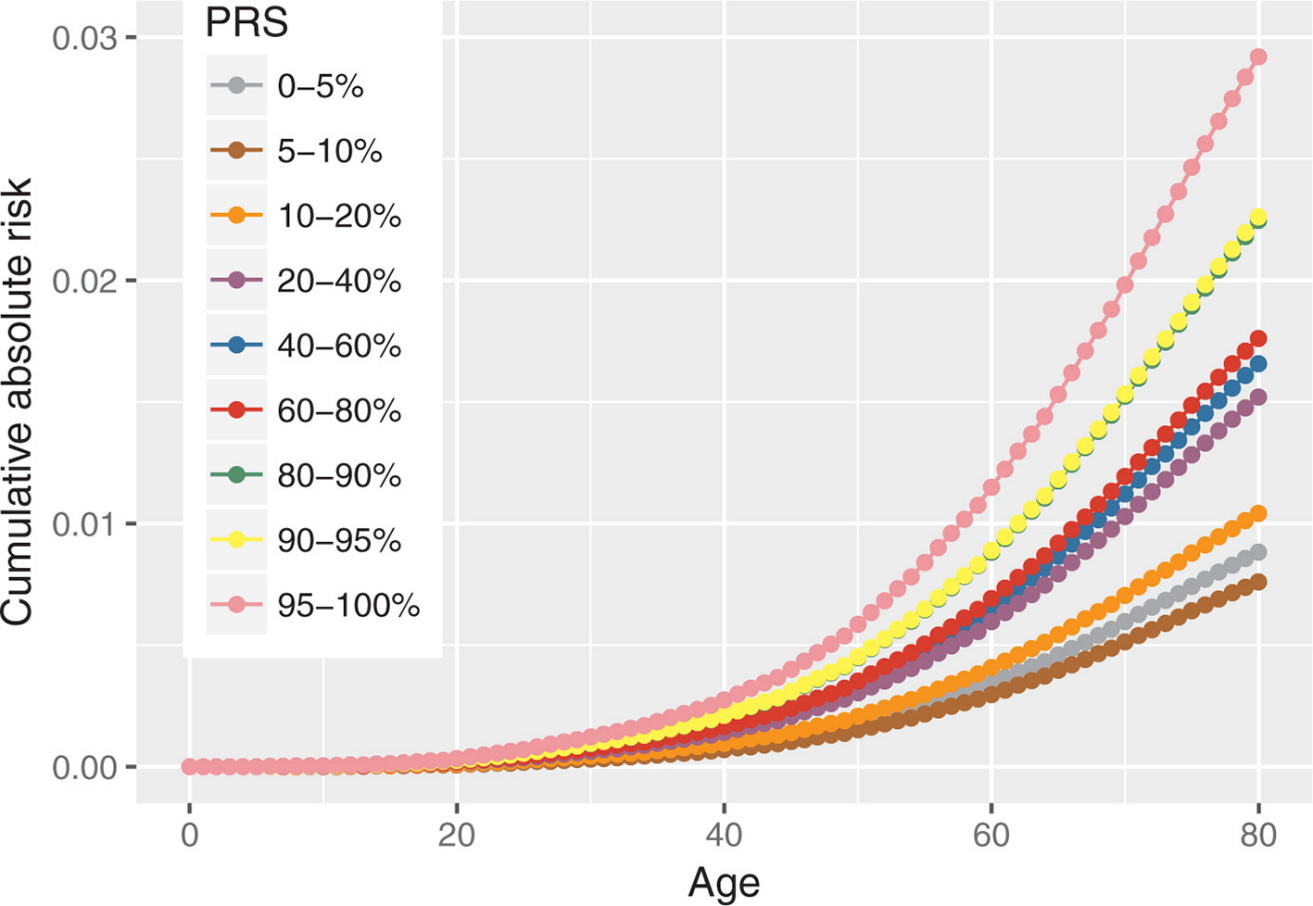
Absolute risk of developing overall ovarian cancers by overall polygenic risk score (PRS) percentiles.

Figure 3 shows the predicted age-specific absolute risk of developing overall EOC by different PRS percentile categories. By age 80, the risks of developing EOC for women in the highest and lowest 5% of the PRS are predicted to be 2.9% and 0.9%, respectively.

## Discussion

Prior to incorporating the effects of common genetic variants into risk prediction models, it is important to calibrate the magnitude of their associations in studies which are independent of the original GWAS. This is the first prospective cohort study to evaluate the combined effects of GWAS identified common SNPs on EOC risk. We used data from a cohort of women in which women with known strong family history of OC or with known high-risk mutations were excluded. Therefore, the results are more applicable to women at ‘low risk’ of developing OC. Our results show that SNPs combine multiplicatively on EOC risk and that the PRS predicts EOC risk prospectively. There was a stronger association with the serous EOC for predicting the risk of serous EOC. This was expected as most of the SNPs used in the PRS construction showed stronger associations with serous EOC in the published GWAS. The empirical OR estimates for EOC associated with different percentiles of the PRS were in line with the theoretical expected values under the multiplicative model, suggesting the PRS is calibrated at the extremes of the distribution, although the OR estimates are associated with wide CIs. There was 3.4-fold difference in the risk of EOC between women at the 5th and 95th percentiles of the PRS. However, the discriminatory ability of the ‘PRS alone’ is modest with an area under the receiver-operating characteristic curve of 0.58–0.60 (based on 1428 controls, 750 overall OC cases, 489 serous OC cases).

There was no evidence of an interaction between the PRS and age in our study, suggesting that the relative effect of the PRS remains constant with age. Little changes were observed in the OR estimates associated with the PRS after adjusting for family history of OC (table 3). Overall, family history of OC was not significantly associated with EOC risk in the present study, but this could be a consequence of the study design. One of the eligibility criteria for inclusion in the UKCTOCS trial was that women had to be at low risk of familial OC; therefore, the overall cohort is ‘biased’ towards women without significant family history of OC. However, the reduction in the effect size for family history after adjusting for the PRS is consistent with the predicted contribution of the SNPs to the familial risk of OC. To assess the effect of family history on OC accurately and the possible attenuation in the family history association after taking into account the PRS, larger studies with more representative samples of women from the population (with respect to family history) would be required.

The current estimate of lifetime EOC risk in the UK general population is 1.86% based on 2014 data. Our results show that using the PRS alone results in a cumulative EOC risk by age 80 of 2.92% for women in the highest 5% of the overall PRS percentiles and 0.88% for women in the lowest 5% of the PRS. Although such differences alone may not lead to changes in the clinical management of women (eg, use of risk-reducing treatments such as salpingo-oophorectomy), the PRS in combination with other established risk factors for EOC such as family history, other known rare genetic susceptibility variants and epidemiological risk factors (eg, OCP use, parity, endometriosis, tubal ligation) is likely to improve EOC risk stratification and help stratify the women in different risk categories.^3 15 16^ For example, under Jervis *et al*’s model,^3^ the risk of developing EOC by age 80 for a woman at the highest 5% of the observed PRS is 6.6% if she has a mother diagnosed with EOC at age 50. Furthermore, by combining all risk factors together, it has been demonstrated that there is a gradient of lifetime risk in unselected populations which ranges from 0.35% to 8.78%.^17^ Although the present study is limited by the inclusion of women with no significant family history of OC, the findings are relevant to tailoring screening efforts in the future. In addition, these PRS can result in clinically significant differences in risk when used in combination with mutations in moderate penetrance genes such as *RAD51C*, *RAD51D* and *BRIP1*.^16^ Hence, risk modelling incorporating a combination of PRS, other rare genetic susceptibility variants, family history and epidemiological factors may enable population risk stratification to identify individuals who will benefit from targeted interventions. For example, risk-reducing salpingo-oophorectomy has been suggested to be cost-effective at >4%–5% lifetime risk of OC.^18 19^ This may provide clinical utility for undertaking surgical prevention above these levels of risk. A change in guidelines to enable women at above these risk thresholds to benefit from surgical prevention has been advocated.^20^

The present study has also several limitations. Some of the study participants had been diagnosed with another cancer prior to their recruitment into the study. These women were included in the analysis (52 incident cases, 111 controls) in line with previous GWAS. After excluding the women with a history of cancer, the results remain similar and the conclusions were not influenced by these assumptions (online supplementary tables 2–4 and supplementary figure 2–5). Although the UKCTOCS study was not included in the recent GWAS,^6^ since this is a national study, it is possible that some incidental overlaps may exist with samples included in the Phelan *et al* study,^6^ if study participants enrolled independently in other studies. On further investigation by the coordinating centre, 34 incident OC cases and 5 of the controls in the study were also included in one of the case–control studies included in Phelan *et al*.^6^ After excluding the overlapping individuals the results remained virtually identical (data not shown). Another limitation is that the majority of the women were eligible to participate in the UKCTOCS study if they did not have family history of OC. Therefore, we were not able to obtain an unbiased estimates of the effect of family history. In the present study, the OR estimate associated with family history of OC is substantially lower compared with studies of familial risks of OC,^3^ but this is expected under the present design. A possible further implication of the present study design (ie, women selected for no family history) is a possible attenuation of the effect of the PRS due to the fact that common variants are expected to be confounded with family history of OC. However, the OR estimates for different percentiles of the PRS were in line with those expected under a multiplicative model, suggesting that this is unlikely to result in a substantial bias in the PRS associations. We note that although the multiplicative model assumption is further supported by the fact that no SNP*SNP interactions were detected, the current study is underpowered to investigate pairwise interactions of modest effects (online supplementary table 5). Larger studies will have to investigate this. The sample size is also limited for assessing the associations of the PRS with different histotypes, other than serous EOC. Although GWAS have demonstrated associations between SNPs and other EOC histotypes, the number of endometrioid, clear cell and mucinous cancers were too small and the PRS did not show evidence associations with these histotypes (online supplementary table 6). Additionally, the latest GWAS has identified many more common SNPs (~30) associated with EOC which were estimated to account for 6.4% of the polygenic risk in the population.^6^ The 15 SNPs included in the PRS and evaluated in the present study explained only 3.4% of the polygenic risk. Therefore, further improvement could be achieved by incorporating these additional SNPs into the PRS or by constructing PRS which include both genomewide significant SNPs and SNPs with higher p values of association using penalised regression models.^21–23^

In conclusion, this paper is the first evaluation of the association of the PRS with EOC in a prospective general population cohort. It demonstrates that the PRS based on published SNP effect sizes is well calibrated and the PRS is a strong risk factor for EOC that contributes towards the discrimination of women who will develop EOC. It will be necessary to incorporate the PRS in comprehensive OC risk prediction models together with other risk factors for the disease and assess the improvement in risk prediction in prospective studies. Such comprehensive risk models will facilitate the clinical decision-making and health management for at-risk women and provide more personalised risk management.
