# Supplementary material for: Evaluation of polygenic risk scores for ovarian cancer risk prediction in a prospective cohort study
Source: J Med Genet. 2018 May 5;55(8):546–54. doi: 10.1136/jmedgenet-2018-105313 (PMC6073911; doi:10.1136/jmedgenet-2018-105313)
Supplement: Supplementary data [file jmedgenet-2018-105313supp001.docx]

**Supplementary material**

**DNA extraction:**

At recruitment, each women donated a blood sample as previously described [3]. Briefly, all samples were transported overnight at ambient temperature to the central laboratory where the blood was centrifuged and serum separated. The serum was pre-cooled at -80°C and stored long-term in liquid nitrogen. DNA was extracted from 500ul of serum using a spin-column based protocol using Qiacube system and QIAamp MinElute Virus Spin Kit (Qiagen, Germany) as per manufacturer’s instructions. DNA quantification was performed using the Picogreen assay (Invitrogen) as per manufacturer’s instructions. The average yield was 233ng.

Supplementary Table 1: The SNPs used in the overall and serous PRS are shown in the first column, which include SNPs that are either identical or in previously published regions. The effect allele frequency (EAF), log odds ratio (logOR) estimate and p-value for each SNP were obtained from the combined iCOGS and OncoArray association analyses of the Ovarian Cancer Association Consortium (OCAC) [2]. The correlation coefficient r^2^ was calculated between the SNPs used in the PRS and the previously published SNPs.

| **SNPs used in the PRS** | **Chr** | **Position^a^** | **Baseline** | **Effect** | **EAF** | **Overall** | | **Serous** | | **Closest Gene** | **r^2^** | **Previously published SNP^b^ demonstrating a genome-wide significant association** |
| --- | --- | --- | --- | --- | --- | --- | --- | --- | --- | --- | --- | --- |
|  |  |  |  |  |  | **logOR** | **p-value** | **logOR** | **p-value** |  |  |  |
| rs58722170 | 1 | 38096421 | G | C | 0.22 | 0.07 | 1.06E-05 | 0.1 | 1.93E-07 | *RSPO1* | 1.00 | rs58722170^c^ |
| rs711830 | 2 | 177037311 | G | A | 0.32 | 0.11 | 3.54E-14 | 0.12 | 2.66E-14 | *HOXD3* | 1.00 | rs711830 |
| rs62274041 | 3 | 156435640 | A | G | 0.95 | -0.37 | 4.45E-38 | -0.46 | 3.28E-46 | *TIPARP* | 1.00 | rs62274041 |
| rs10069690 | 5 | 1279790 | G | A | 0.26 | 0.08 | 3.42E-08 | 0.12 | 1.48E-12 | *TERT* | 1.00 | rs10069690 |
| rs78724141 | 8 | 82659661 | G | T | 0.07 | 0.15 | 1.81E-09 | 0.19 | 1.40E-10 | *CHMP4C* | 0.96 | rs76837345 |
| rs10088218 | 8 | 129543949 | G | A | 0.13 | -0.16 | 9.27E-16 | -0.24 | 1.90E-24 | *CMYC/PVT1* | 1.00 | rs1400482 |
| rs7032221 | 9 | 16914895 | A | G | 0.32 | -0.18 | 1.40E-35 | -0.22 | 9.21E-41 | *BNC2* | 0.51 | rs10962692 |
| rs635634 | 9 | 136155000 | C | T | 0.2 | 0.1 | 3.08E-09 | 0.12 | 1.26E-10 | *ABO* | 0.98 | 9:136138765 |
| rs1802669 | 10 | 21827796 | G | A | 0.35 | 0.08 | 2.00E-09 | 0.09 | 7.10E-09 | *MLLT10* | 0.83 | rs144962376 |
| rs7135337 | 12 | 121404155 | A | C | 0.58 | -0.06 | 7.19E-06 | -0.08 | 3.99E-07 | *HNF1A-AS1* | 0.94 | rs7953249^c^ |
| rs11651755 | 17 | 36099840 | A | G | 0.49 | 0.03 | 1.77E-02 | 0.09 | 1.40E-08 | *HNF1B* | 0.54 | rs7405776 |
| rs1105569 | 17 | 43793388 | C | T | 0.22 | 0.11 | 9.04E-13 | 0.12 | 7.72E-11 | *PLEKHM1* | 0.76 | rs1879586 |
| rs7207826 | 17 | 46500673 | T | C | 0.27 | 0.1 | 7.67E-13 | 0.13 | 1.15E-14 | *SKAP1* | 1.00 | rs7207826 |
| rs61494113 | 19 | 17401859 | G | A | 0.3 | 0.12 | 6.00E-17 | 0.16 | 2.55E-23 | *BABAM1* | 0.99 | rs4808075 |
| rs9625477 | 22 | 28858248 | T | C | 0.1 | -0.1 | 3.76E-06 | -0.15 | 1.69E-08 | *TTC28/CHEK2* | 0.95 | rs6005807 |

^a^The SNP position is based on GRCh37.

^b^SNPs from [2].

^c^SNPs demonstrated association at genome-wide signal levels in the meta-analysis combining OCAC and CIMBA data (p-value = 1.4E-09 for rs58722170; p-value = 4.5E-10 for rs7953249) [2].

Supplementary Table 2: A summary of epidemiological characteristics of the 2015 subjects who were unaffected at the recruitment.

|  | | **Controls** | **Cases** | **P_difference^1^** |
| --- | --- | --- | --- | --- |
| **Number of women** |  | 1317 | 698 |  |
| **Age at baseline (%)** | | | | 0.42 |
| <60 |  | 467 (35.5%) | 243 (34.8%) |  |
| 60-69 |  | 636 (48.3%) | 355 (50.9%) |  |
| ≥70 |  | 214 (16.2%) | 100 (14.3%) |  |
| **Age at censoring (%)** | | | | <.0001 |
| <60 |  | 15 (1.1%) | 102 (14.6%) |  |
| 60-69 |  | 343 (26.1%) | 327 (46.8%) |  |
| 70-79 |  | 653 (49.6%) | 255 (36.5%) |  |
| ≥80 |  | 306 (23.2%) | 14 (2.1%) |  |
| **Birth cohort (%)** | | | | 0.73 |
| <1930 |  | 26 (2.0%) | 9 (1.3%) |  |
| 1930-1939 |  | 585 (44.4%) | 312 (44.7%) |  |
| 1940-1949 |  | 620 (47.1%) | 333 (47.7%) |  |
| ≥1950 |  | 86 (6.5%) | 44 (6.3%) |  |
| **Mean age at baseline (sd)** |  | 63 (6.2) | 63 (6.1) |  |
| **Mean censored age (sd)** |  | 74 (6.5) | 68 (6.6) |  |
| **Mean PRS (sd)** | | | |  |
|  | Overall | -0.47 (0.27) | -0.39 (0.27) |  |
|  | Serous | -0.56 (0.35) | -0.43 (0.35) |  |
| **Family history of ovarian cancer (%)** | | | |  |
| Considering only 1^st^ degree relatives | 0 affected relatives | 1278 (97.0%) | 668 (95.7%) |  |
|  | 1 affected relative | 39 (3.0%) | 30 (4.3%) | 0.12 |
| Considering both 1^st^ and 2^nd^ degree | 0 affected relatives | 1250 (94.9%) | 653 (93.6%) |  |
|  | ≥1 affected relatives | 67 (5.1%) | 45 (6.4%) | 0.21 |
| **Morphology/histotype (N)** | | | |  |
| Serous |  |  | 452 |  |
|  | High grade |  | 381 |  |
|  | Low grade |  | 23 |  |
|  | Missing |  | 48 |  |
| Clear cell |  |  | 28 |  |
| Endometrioid |  |  | 54 |  |
| Mucinous |  |  | 23 |  |
| Others |  |  | 141 |  |

1: Chi-square tests for differences in the distributions between cases and controls.

Supplementary Table 3: Association between PRS and ovarian cancer in different age groups using 2015 subjects who were unaffected at the recruitment. In the overall PRS analysis we used cases of any type of ovarian cancers and in the serous PRS analysis we used cases of serous ovarian cancer.

| **Age group** | **Overall** | | **Serous** | |
| --- | --- | --- | --- | --- |
|  | **OR (95% CI)** | **p-value** | **OR (95% CI)** | **p-value** |
| **All ages** | 1.32 (1.20-1.45) | 4.46×10^-9^ | 1.44 (1.29-1.60) | 3.02×10^-11^ |
| **<60** | 1.64 (0.86-3.33) | 0.15 | 2.16 (1.08-.91) | 0.04 |
| **60-69** | 1.28 (1.10-1.49) | 1.98×10^-3^ | 1.35 (1.14-1.61) | 6.51×10^-4^ |
| **≥70** | 1.36 (1.17-1.53) | 2.33×10^-5^ | 1.46 (1.25-1.71) | 2.54×10^-6^ |
| **Interaction** | 1.01 (0.99-1.02) | 0.55 | 1.01 (0.99-1.03) | 0.61 |

Supplementary Table 4: Association between PRS percentiles and ovarian cancer risk: unadjusted and adjusted by family history of ovarian cancer in 1^st^ degree or in 1^st^ and 2^nd^ degree relatives using 2015 subjects who were unaffected at the recruitment.

**(a) Overall**

| **PRS percentile**  **category (%)** | **Number of**  **Controls** | **Number of**  **Overall cases** | **OR (95% CI)** | | | |
| --- | --- | --- | --- | --- | --- | --- |
|  |  |  | **Unadjusted by FH*** | **Adjusted by 1^st^ degree FH** | **Adjusted by 1^st^ & 2^nd^ degree FH** | |
| **[0,5)** | 66 | 19 | 0.55 (0.31-0.93) | 0.55 (0.31-0.94) | 0.55 (0.31-0.95) | |
| **[5,10)** | 66 | 15 | 0.43 (0.23-0.77) | 0.44 (0.23-0.77) | 0.47 (0.23-0.77) | |
| **[10,20)** | 132 | 45 | 0.65 (0.43-0.96) | 0.65 (0.43-0.96) | 0.64 (0.44-0.97) | |
| **[20,40)** | 263 | 126 | 0.91 (0.68-1.23) | 0.92 (0.68-1.23) | 0.92 (0.68-1.24) | |
| **[40,60)** | 263 | 138 | 1 | 1 | 1 | |
| **[60,80)** | 263 | 153 | 1.11 (0.83-1.48) | 1.11 (0.84-1.48) | 1.09 (0.83-1.48) | |
| **[80,90)** | 132 | 98 | 1.41 (1.01-1.97) | 1.42 (1.02-1.98) | 1.42 (1.02-1.99) | |
| **[90,95)** | 66 | 42 | 1.21 (0.78-1.87) | 1.21 (0.77-1.87) | 1.32 (0.78-1.87) | |
| **[95,100]** | 66 | 62 | 1.79 (1.20-2.68) | 1.81 (1.21-2.70) | 1.71 (1.20-2.69) | |
| **FH** |  |  |  | 1.49 (0.90-2.42) | 1.27 (0.85-1.86) | |
| **(b) Serous** | | | | | |  |
| **PRS percentile**  **category (%)** | **Number of**  **Controls** | **Number of**  **Serous cases** | **OR (95% CI)** | | | |
|  |  |  | **Unadjusted by FH** | **Adjusted by 1^st^ degree FH** | **Adjusted by 1^st^ & 2^nd^ degree FH** | |
| **[0,5)** | 66 | 10 | 0.46 (0.22-0.90) | 0.46 (0.22-0.91) | 0.47 (0.22-0.91) | |
| **[5,10)** | 66 | 9 | 0.42 (0.19-0.83) | 0.41 (0.19-0.83) | 0.42 (0.19-0.83) | |
| **[10,20)** | 132 | 29 | 0.67 (0.41-1.06) | 0.68 (0.42-1.07) | 0.68 (0.42-1.07) | |
| **[20,40)** | 263 | 73 | 0.85 (0.59-1.21) | 0.85 (0.59-1.21) | 0.85 (0.60-1.21) | |
| **[40,60)** | 263 | 86 | 1 | 1 | 1 | |
| **[60,80)** | 263 | 97 | 1.13 (0.81-1.58) | 1.13 (0.81-1.58 | 1.13 (0.81-1.58) | |
| **[80,90)** | 132 | 66 | 1.53 (1.04-2.24) | 1.53 (1.04-2.24) | 1.53 (1.04-2.25) | |
| **[90,95)** | 66 | 34 | 1.58 (0.97-2.54) | 1.59 (0.97-2.55) | 1.59 (0.97-2.55) | |
| **[95,100]** | 66 | 48 | 2.22 (1.42-3.47) | 2.23 (1.42-3.47) | 2.22 (1.42-3.46) | |
| **FH** |  |  |  | 1.45 (0.81-2.53) | 1.28 (0.81-2.00) | |

*FH=family history of ovarian cancer

Supplementary Table 5: Examples of power calculations for pair-wise SNP*SNP interaction analysis†.

| SNP1 | | | SNP2 | | | Power to detect interaction at OR: | | | |
| --- | --- | --- | --- | --- | --- | --- | --- | --- | --- |
| SNP | Allele frequency | Per-allele OR | SNP | Allele frequency | Per-allele OR | 1.05 | 1.1 | 1.15 | 1.2 |
| rs58722170 | 0.22 | 1.07 | rs711830 | 0.32 | 1.12 | 7.2% | 13.5% | 23.7% | 36.8% |
| rs10069690 | 0.26 | 1.08 | rs7032221 | 0.32 | 0.84 | 7.3% | 13.9% | 24.6% | 38.3% |
| rs78724141 | 0.07 | 1.16 | rs10088218 | 0.13 | 0.85 | 5.4% | 6.6% | 8.5% | 11.1% |
| rs1802669 | 0.35 | 1.08 | rs7135337 | 0.58 | 0.94 | 8.1% | 17.3% | 31.7% | 49.0% |
| rs11651755 | 0.49 | 1.03 | rs1105569 | 0.22 | 1.12 | 7.5% | 14.7% | 26.1% | 40.5% |
| rs7032221 | 0.32 | 0.84 | rs10088218 | 0.13 | 0.85 | 6.2% | 9.7% | 15.3% | 23.0% |
| rs9625477 | 0.1 | 0.90 | rs1165175 | 0.49 | 1.03 | 6.2% | 9.5% | 14.9% | 22.1% |
| rs9625477 | 0.1 | 0.90 | rs10069690 | 0.26 | 1.08 | 5.9% | 8.6% | 13.0% | 18.9% |

†Power was calculated using the QUANTO[4, 5]. Parameters assumed were allele frequency and OR for the marginal effect of each SNP, based on a sample of 750 cases with 2 controls for each case. Power was calculated for a significance level 0.05, and assuming ovarian cancer population prevalence of 0.019.

Supplementary Table 6: Association between PRS and different histotypes of ovarian cancer using all the 2178 subjects (including those with cancer history before recruitment) using multinomial regression analysis.

| **Histotype** | **OR (95% CI)** | **p-value** |
| --- | --- | --- |
| Serous | 1.43 (1.29-1.59) | 5.91×10^-12^ |
| Clear cell | 0.82 (0.56-1.20) | 0.30 |
| Endometrioid | 0.89 (0.68-1.18) | 0.43 |
| Mucinous | 0.90 (0.59-1.36) | 0.61 |
| Others | 1.37 (1.16-1.61) | 1.69×10^-4^ |

Supplementary Figure 1: Selection of SNPs for genotyping and for inclusion in the final PRS.

**96 SNPs selected for genotyping**

on the basis of association results from the iCOGS experiment [1] in 2015. Selected top SNPs and other correlated (proxy SNPs) from regions that demonstrated associations at p< 10^-5^.

52 SNPs failed quality control

**44 SNPs**

Phelan et al. [2] in 2017, provided updated GWAS results that established 27 regions with ~30 SNPs showing genome-wide significant results in either overall ovarian cancer or any subtypes. This updated publication was used to refine the genome-wide significant regions.

**25 SNPs**

in non-genome-wide significant regions

**19 SNPs**

in 15 genome-wide significant regions

selected the top SNP in each region

**15 SNPs**

used for analysis, Supplementary Table 1

**Excluded**

Supplementary Figure 2: QQplot shows the observed against expected −log_10_P values of pair-wise SNP*SNP interaction tests under the null hypothesis of multiplicative model using 2015 subjects who were unaffected at the recruitment. The dashed line shows the 95% concentration band.


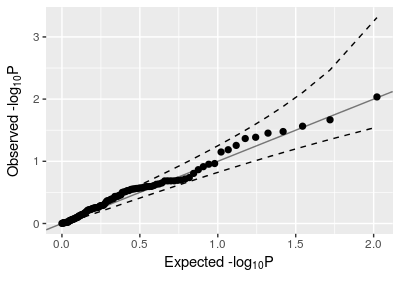


Supplementary Figure 3: Distribution of the standardized overall and serous PRS in overall and serous ovarian cancer cases and controls using 2015 subjects who were unaffected at the recruitment. The dashed vertical lines show the PRS means.


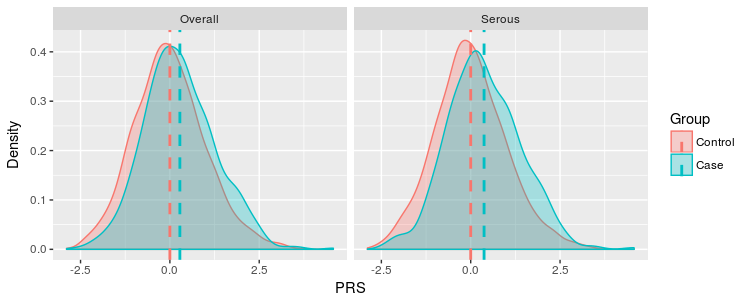


Supplementary Figure 4: Odds ratio estimates between overall/serous PRS percentiles and overall/serous ovarian cancer risk relative to the middle PRS quintile (40-60%) using 2015 subjects who were unaffected at the recruitment. The solid line shows the estimated ORs with 95% CI and the dashed line represents the theoretical OR values assuming multiplicative model.


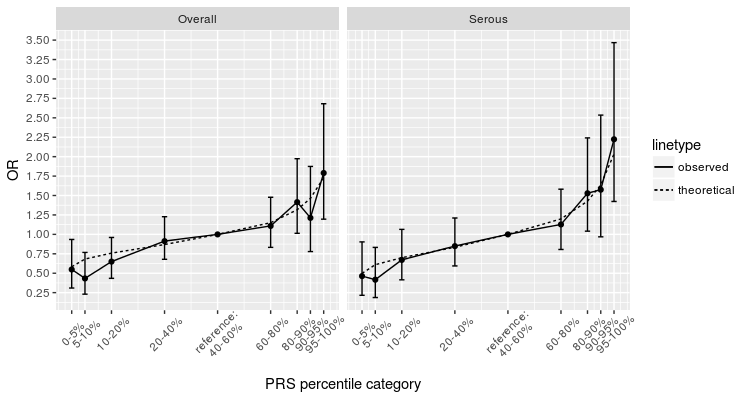


Supplementary Figure 5: Cumulative absolute risk of developing overall ovarian cancers by different overall PRS percentile using relative risks estimated from 2015 subjects who were unaffected at the recruitment.


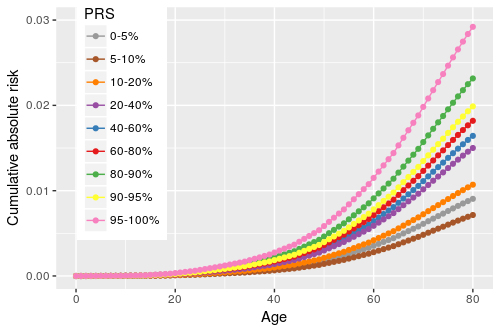


**Reference:**

1. Kuchenbaecker KB, Ramus SJ, Tyrer J, Lee A, Shen HC, Beesley J, Lawrenson K, McGuffog L, Healey S, Lee JM, Spindler TJ, Lin YG, Pejovic T, Bean Y, Li Q, Coetzee S, Hazelett D, Miron A, Southey M, Terry MB, Goldgar DE, Buys SS, Janavicius R, Dorfling CM, van Rensburg EJ, Neuhausen SL, Ding YC, Hansen TVO, Jønson L, Gerdes A-M, Ejlertsen B, Barrowdale D, Dennis J, Benitez J, Osorio A, Garcia MJ, Komenaka I, Weitzel JN, Ganschow P, Peterlongo P, Bernard L, Viel A, Bonanni B, Peissel B, Manoukian S, Radice P, Papi L, Ottini L, Fostira F, Konstantopoulou I, Garber J, Frost D, Perkins J, Platte R, Ellis S, Godwin AK, Schmutzler RK, Meindl A, Engel C, Sutter C, Sinilnikova OM, Damiola F, Mazoyer S, Stoppa-Lyonnet D, Claes K, De Leeneer K, Kirk J, Rodriguez GC, Piedmonte M, O’Malley DM, de la Hoya M, Caldes T, Aittomäki K, Nevanlinna H, Collée JM, Rookus MA, Oosterwijk JC, Tihomirova L, Tung N, Hamann U, Isaccs C, Tischkowitz M, Imyanitov EN, Caligo MA, Campbell IG, Hogervorst FBL, Olah E, Diez O, Blanco I, Brunet J, Lazaro C, Pujana MA, Jakubowska A, Gronwald J, Lubinski J, Sukiennicki G, Barkardottir RB, Plante M, Simard J, Soucy P, Montagna M, Tognazzo S, Teixeira MR, Pankratz VS, Wang X, Lindor N, Szabo CI, Kauff N, Vijai J, Aghajanian CA, Pfeiler G, Berger A, Singer CF, Tea M-K, Phelan CM, Greene MH, Mai PL, Rennert G, Mulligan AM, Tchatchou S, Andrulis IL, Glendon G, Toland AE, Jensen UB, Kruse TA, Thomassen M, Bojesen A, Zidan J, Friedman E, Laitman Y, Soller M, Liljegren A, Arver B, Einbeigi Z, Stenmark-Askmalm M, Olopade OI, Nussbaum RL, Rebbeck TR, Nathanson KL, Domchek SM, Lu KH, Karlan BY, Walsh C, Lester J, Hein A, Ekici AB, Beckmann MW, Fasching PA, Lambrechts D, Van Nieuwenhuysen E, Vergote I, Lambrechts S, Dicks E, Doherty JA, Wicklund KG, Rossing MA, Rudolph A, Chang-Claude J, Wang-Gohrke S, Eilber U, Moysich KB, Odunsi K, Sucheston L, Lele S, Wilkens LR, Goodman MT, Thompson PJ, Shvetsov YB, Runnebaum IB, Dürst M, Hillemanns P, Dörk T, Antonenkova N, Bogdanova N, Leminen A, Pelttari LM, Butzow R, Modugno F, Kelley JL, Edwards RP, Ness RB, du Bois A, Heitz F, Schwaab I, Harter P, Matsuo K, Hosono S, Orsulic S, Jensen A, Kjaer SK, Hogdall E, Hasmad HN, Azmi MAN, Teo S-H, Woo Y-L, Fridley BL, Goode EL, Cunningham JM, Vierkant RA, Bruinsma F, Giles GG, Liang D, Hildebrandt MAT, Wu X, Levine DA, Bisogna M, Berchuck A, Iversen ES, Schildkraut JM, Concannon P, Weber RP, Cramer DW, Terry KL, Poole EM, Tworoger SS, Bandera EV, Orlow I, Olson SH, Krakstad C, Salvesen HB, Tangen IL, Bjorge L, van Altena AM, Aben KKH, Kiemeney LA, Massuger LFAG, Kellar M, Brooks-Wilson A, Kelemen LE, Cook LS, Le ND, Cybulski C, Yang H, Lissowska J, Brinton LA, Wentzensen N, Hogdall C, Lundvall L, Nedergaard L, Baker H, Song H, Eccles D, McNeish I, Paul J, Carty K, Siddiqui N, Glasspool R, Whittemore AS, Rothstein JH, McGuire V, Sieh W, Ji B-T, Zheng W, Shu X-O, Gao Y-T, Rosen B, Risch HA, McLaughlin JR, Narod SA, Monteiro AN, Chen A, Lin H-Y, Permuth-Wey J, Sellers TA, Tsai Y-Y, Chen Z, Ziogas A, Anton-Culver H, Gentry-Maharaj A, Menon U, Harrington P, Lee AW, Wu AH, Pearce CL, Coetzee G, Pike MC, Dansonka-Mieszkowska A, Timorek A, Rzepecka IK, Kupryjanczyk J, Freedman M, Noushmehr H, Easton DF, Offit K, Couch FJ, Gayther S, Pharoah PP, Antoniou AC, Chenevix-Trench G, Easton DF, Offit K, Couch FJ, Gayther S, Pharoah PP, Antoniou AC, Chenevix-Trench G, Brca tCoIoMoB. Identification of six new susceptibility loci for invasive epithelial ovarian cancer. *Nature Genetics*. 2015;47(2):164-71.

2. Phelan CM, Kuchenbaecker KB, Tyrer JP, Kar SP, Lawrenson K, Winham SJ, Dennis J, Pirie A, Riggan MJ, Chornokur G, Earp MA, Lyra PC, Jr., Lee JM, Coetzee S, Beesley J, McGuffog L, Soucy P, Dicks E, Lee A, Barrowdale D, Lecarpentier J, Leslie G, Aalfs CM, Aben KK, Adams M, Adlard J, Andrulis IL, Anton-Culver H, Antonenkova N, group As, Aravantinos G, Arnold N, Arun BK, Arver B, Azzollini J, Balmana J, Banerjee SN, Barjhoux L, Barkardottir RB, Bean Y, Beckmann MW, Beeghly-Fadiel A, Benitez J, Bermisheva M, Bernardini MQ, Birrer MJ, Bjorge L, Black A, Blankstein K, Blok MJ, Bodelon C, Bogdanova N, Bojesen A, Bonanni B, Borg A, Bradbury AR, Brenton JD, Brewer C, Brinton L, Broberg P, Brooks-Wilson A, Bruinsma F, Brunet J, Buecher B, Butzow R, Buys SS, Caldes T, Caligo MA, Campbell I, Cannioto R, Carney ME, Cescon T, Chan SB, Chang-Claude J, Chanock S, Chen XQ, Chiew YE, Chiquette J, Chung WK, Claes KB, Conner T, Cook LS, Cook J, Cramer DW, Cunningham JM, D'Aloisio AA, Daly MB, Damiola F, Damirovna SD, Dansonka-Mieszkowska A, Dao F, Davidson R, DeFazio A, Delnatte C, Doheny KF, Diez O, Ding YC, Doherty JA, Domchek SM, Dorfling CM, Dork T, Dossus L, Duran M, Durst M, Dworniczak B, Eccles D, Edwards T, Eeles R, Eilber U, Ejlertsen B, Ekici AB, Ellis S, Elvira M, Study E, Eng KH, Engel C, Evans DG, Fasching PA, Ferguson S, Ferrer SF, Flanagan JM, Fogarty ZC, Fortner RT, Fostira F, Foulkes WD, Fountzilas G, Fridley BL, Friebel TM, Friedman E, Frost D, Ganz PA, Garber J, Garcia MJ, Garcia-Barberan V, Gehrig A, Collaborators GS, Gentry-Maharaj A, Gerdes AM, Giles GG, Glasspool R, Glendon G, Godwin AK, Goldgar DE, Goranova T, Gore M, Greene MH, Gronwald J, Gruber S, Hahnen E, Haiman CA, Hakansson N, Hamann U, Hansen TV, Harrington PA, Harris HR, Hauke J, Study H, Hein A, Henderson A, Hildebrandt MA, Hillemanns P, Hodgson S, Hogdall CK, Hogdall E, Hogervorst FB, Holland H, Hooning MJ, Hosking K, Huang RY, Hulick PJ, Hung J, Hunter DJ, Huntsman DG, Huzarski T, Imyanitov EN, Isaacs C, Iversen ES, Izatt L, Izquierdo A, Jakubowska A, James P, Janavicius R, Jernetz M, Jensen A, Jensen UB, John EM, Johnatty S, Jones ME, Kannisto P, Karlan BY, Karnezis A, Kast K, Investigators KC, Kennedy CJ, Khusnutdinova E, Kiemeney LA, Kiiski JI, Kim SW, Kjaer SK, Kobel M, Kopperud RK, Kruse TA, Kupryjanczyk J, Kwong A, Laitman Y, Lambrechts D, Larranaga N, Larson MC, Lazaro C, Le ND, Le Marchand L, Lee JW, Lele SB, Leminen A, Leroux D, Lester J, Lesueur F, Levine DA, Liang D, Liebrich C, Lilyquist J, Lipworth L, Lissowska J, Lu KH, Lubinnski J, Luccarini C, Lundvall L, Mai PL, Mendoza-Fandino G, Manoukian S, Massuger LF, May T, Mazoyer S, McAlpine JN, McGuire V, McLaughlin JR, McNeish I, Meijers-Heijboer H, Meindl A, Menon U, Mensenkamp AR, Merritt MA, Milne RL, Mitchell G, Modugno F, Moes-Sosnowska J, Moffitt M, Montagna M, Moysich KB, Mulligan AM, Musinsky J, Nathanson KL, Nedergaard L, Ness RB, Neuhausen SL, Nevanlinna H, Niederacher D, Nussbaum RL, Odunsi K, Olah E, Olopade OI, Olsson H, Olswold C, O'Malley DM, Ong KR, Onland-Moret NC, group Os, Orr N, Orsulic S, Osorio A, Palli D, Papi L, Park-Simon TW, Paul J, Pearce CL, Pedersen IS, Peeters PH, Peissel B, Peixoto A, Pejovic T, Pelttari LM, Permuth JB, Peterlongo P, Pezzani L, Pfeiler G, Phillips KA, Piedmonte M, Pike MC, Piskorz AM, Poblete SR, Pocza T, Poole EM, Poppe B, Porteous ME, Prieur F, Prokofyeva D, Pugh E, Pujana MA, Pujol P, Radice P, Rantala J, Rappaport-Fuerhauser C, Rennert G, Rhiem K, Rice P, Richardson A, Robson M, Rodriguez GC, Rodriguez-Antona C, Romm J, Rookus MA, Rossing MA, Rothstein JH, Rudolph A, Runnebaum IB, Salvesen HB, Sandler DP, Schoemaker MJ, Senter L, Setiawan VW, Severi G, Sharma P, Shelford T, Siddiqui N, Side LE, Sieh W, Singer CF, Sobol H, Song H, Southey MC, Spurdle AB, Stadler Z, Steinemann D, Stoppa-Lyonnet D, Sucheston-Campbell LE, Sukiennicki G, Sutphen R, Sutter C, Swerdlow AJ, Szabo CI, Szafron L, Tan YY, Taylor JA, Tea MK, Teixeira MR, Teo SH, Terry KL, Thompson PJ, Thomsen LC, Thull DL, Tihomirova L, Tinker AV, Tischkowitz M, Tognazzo S, Toland AE, Tone A, Trabert B, Travis RC, Trichopoulou A, Tung N, Tworoger SS, van Altena AM, Van Den Berg D, van der Hout AH, van der Luijt RB, Van Heetvelde M, Van Nieuwenhuysen E, van Rensburg EJ, Vanderstichele A, Varon-Mateeva R, Vega A, Edwards DV, Vergote I, Vierkant RA, Vijai J, Vratimos A, Walker L, Walsh C, Wand D, Wang-Gohrke S, Wappenschmidt B, Webb PM, Weinberg CR, Weitzel JN, Wentzensen N, Whittemore AS, Wijnen JT, Wilkens LR, Wolk A, Woo M, Wu X, Wu AH, Yang H, Yannoukakos D, Ziogas A, Zorn KK, Narod SA, Easton DF, Amos CI, Schildkraut JM, Ramus SJ, Ottini L, Goodman MT, Park SK, Kelemen LE, Risch HA, Thomassen M, Offit K, Simard J, Schmutzler RK, Hazelett D, Monteiro AN, Couch FJ, Berchuck A, Chenevix-Trench G, Goode EL, Sellers TA, Gayther SA, Antoniou AC, Pharoah PD. Identification of 12 new susceptibility loci for different histotypes of epithelial ovarian cancer. *Nat Genet*. 2017.

3. Menon U, Gentry-Maharaj A, Ryan A, Sharma A, Burnell M, Hallett R, Lewis S, Lopez A, Godfrey K, Oram D, Herod J, Williamson K, Seif M, Scott I, Mould T, Woolas R, Murdoch J, Dobbs S, Amso N, Leeson S, Cruickshank D, McGuire A, Campbell S, Fallowfield L, Skates S, Parmar M, Jacobs I. Recruitment to multicentre trials--lessons from UKCTOCS: descriptive study. *BMJ (Clinical research ed)*. 2008;337:a2079.

4. Gauderman WJ. Sample size requirements for association studies of gene-gene interaction. *Am J Epidemiol*. 2002;155(5):478-84.

5. Gauderman WJ, Morrison JM. QUANTO 1.1: A computerprogram for power and sample size calculations for genetic-epidemiology studies. 2006.
